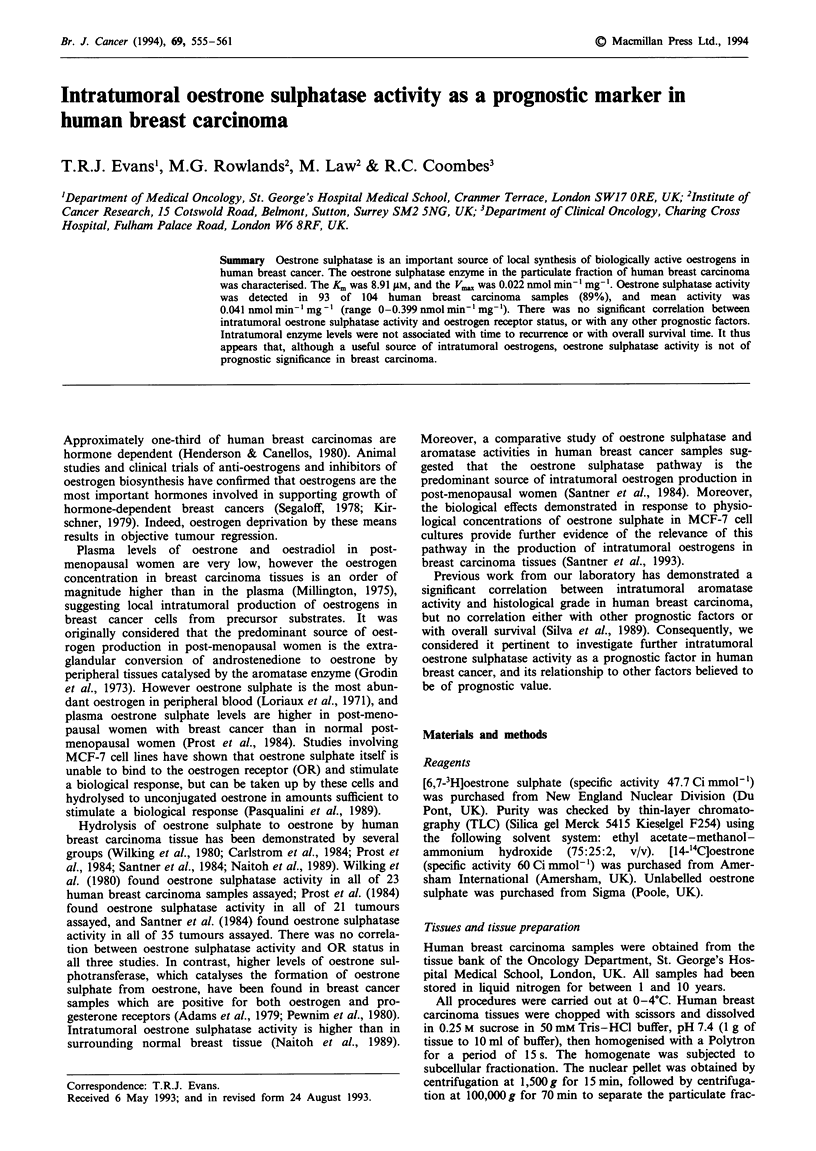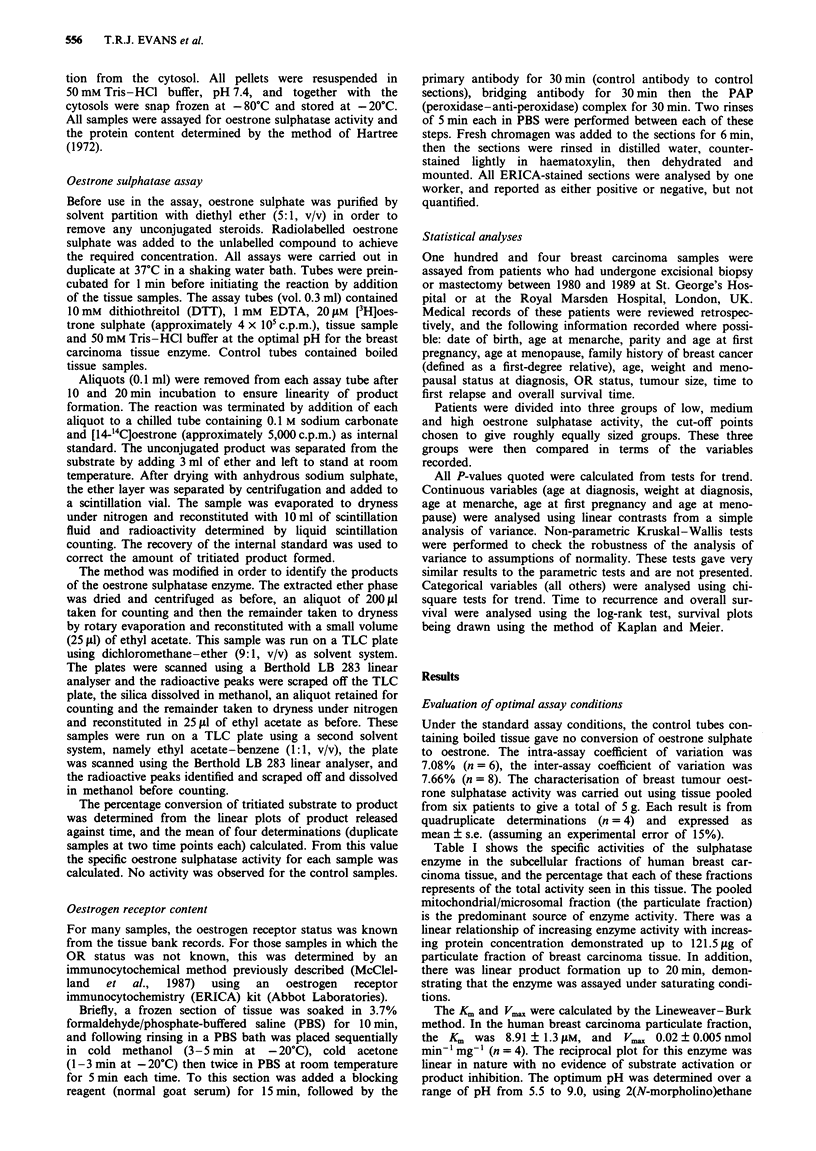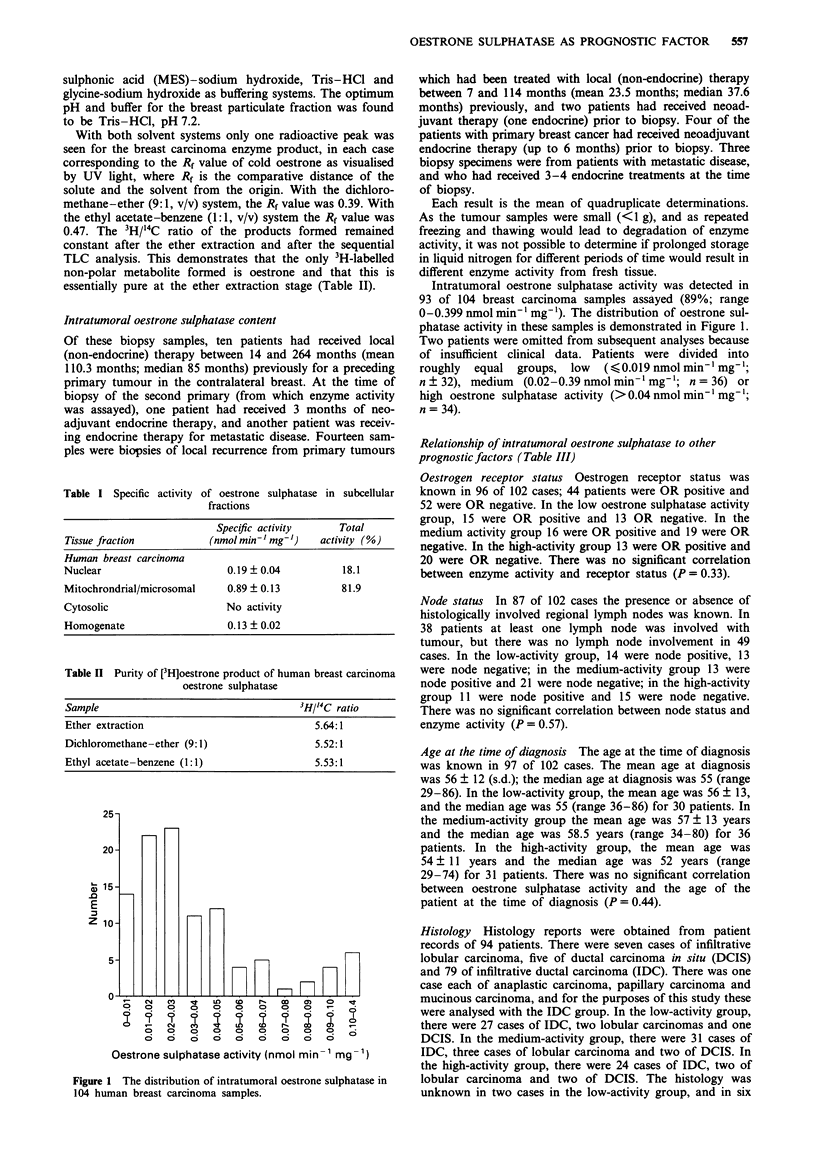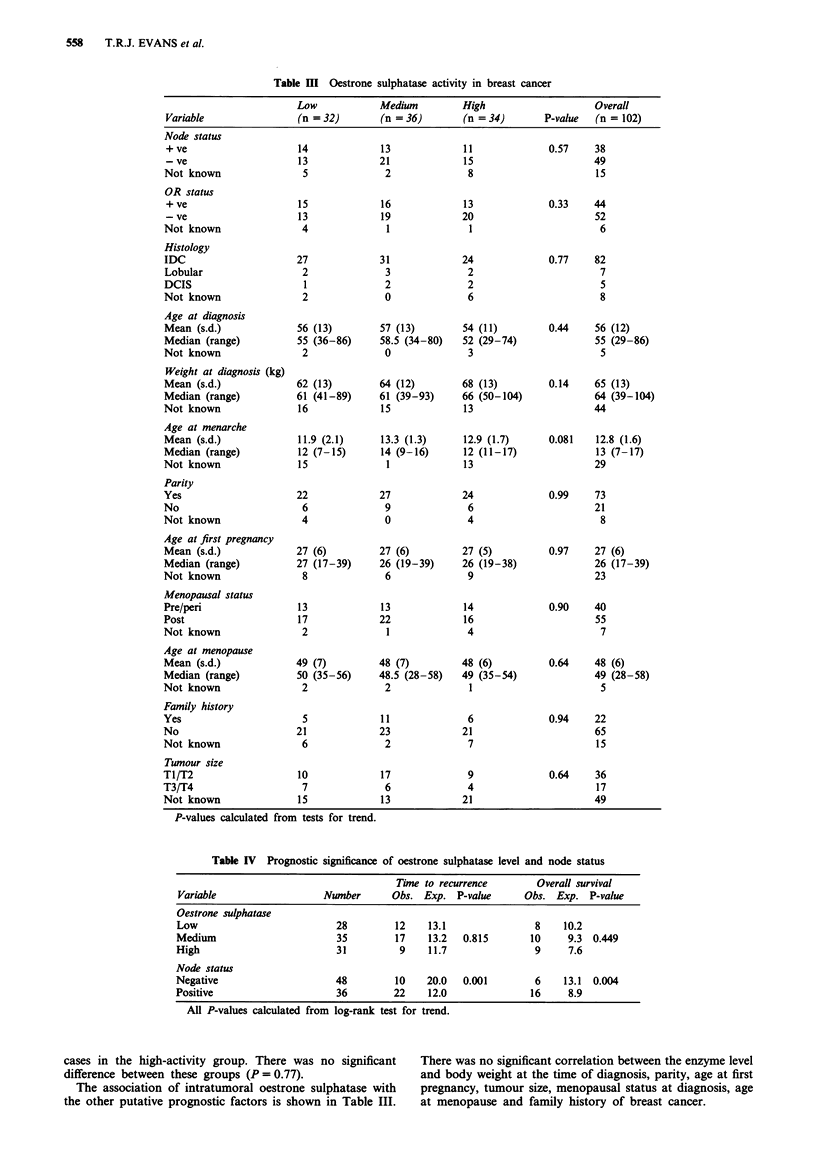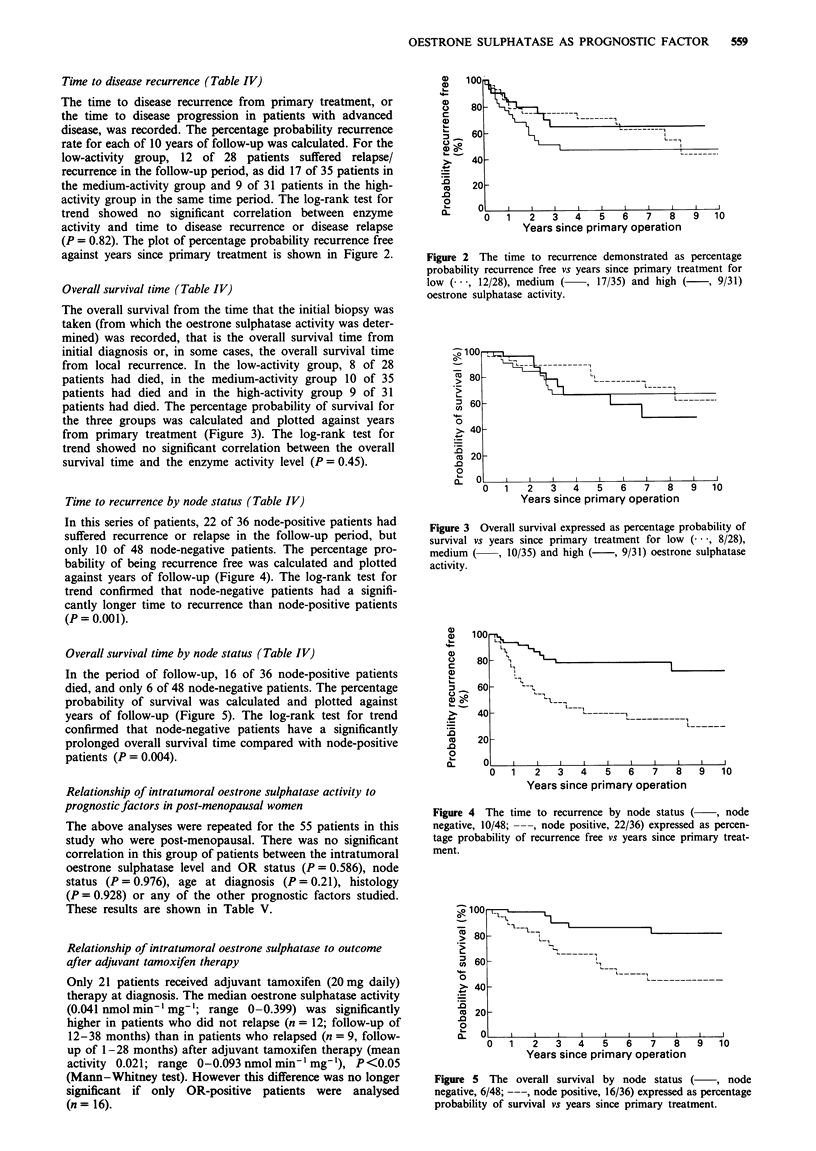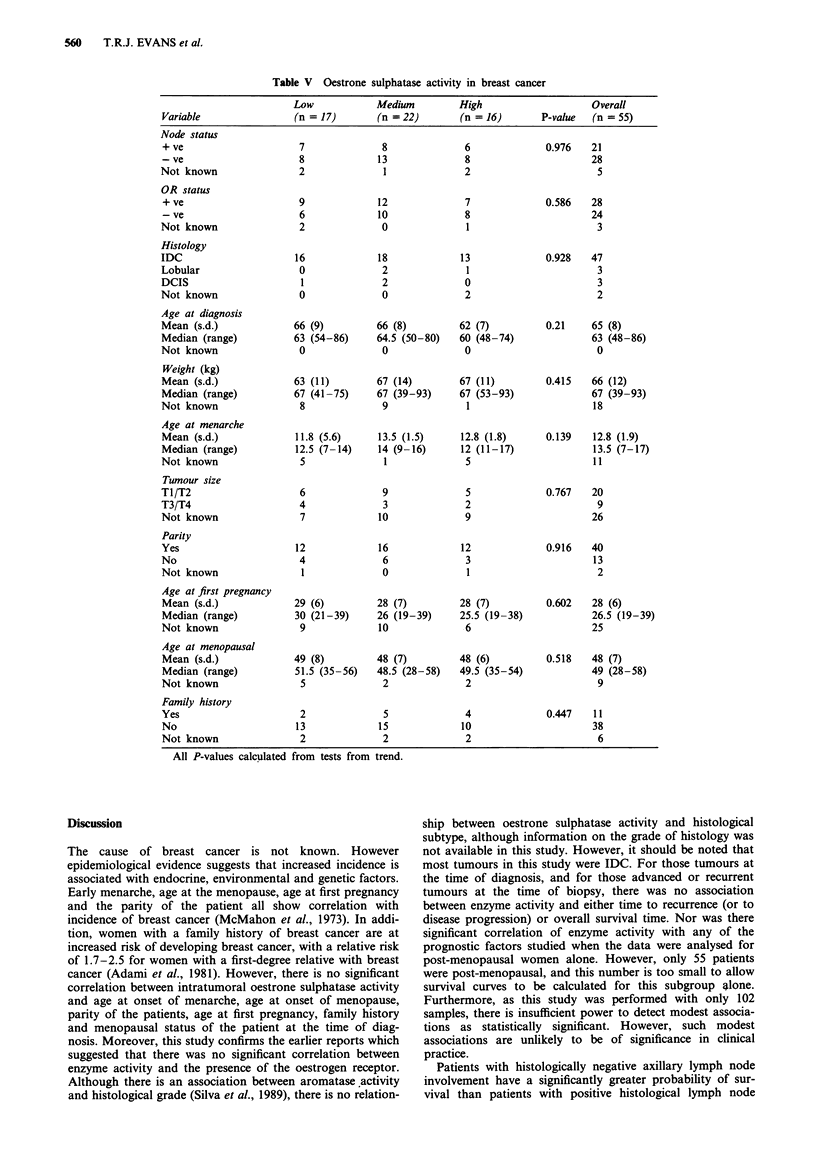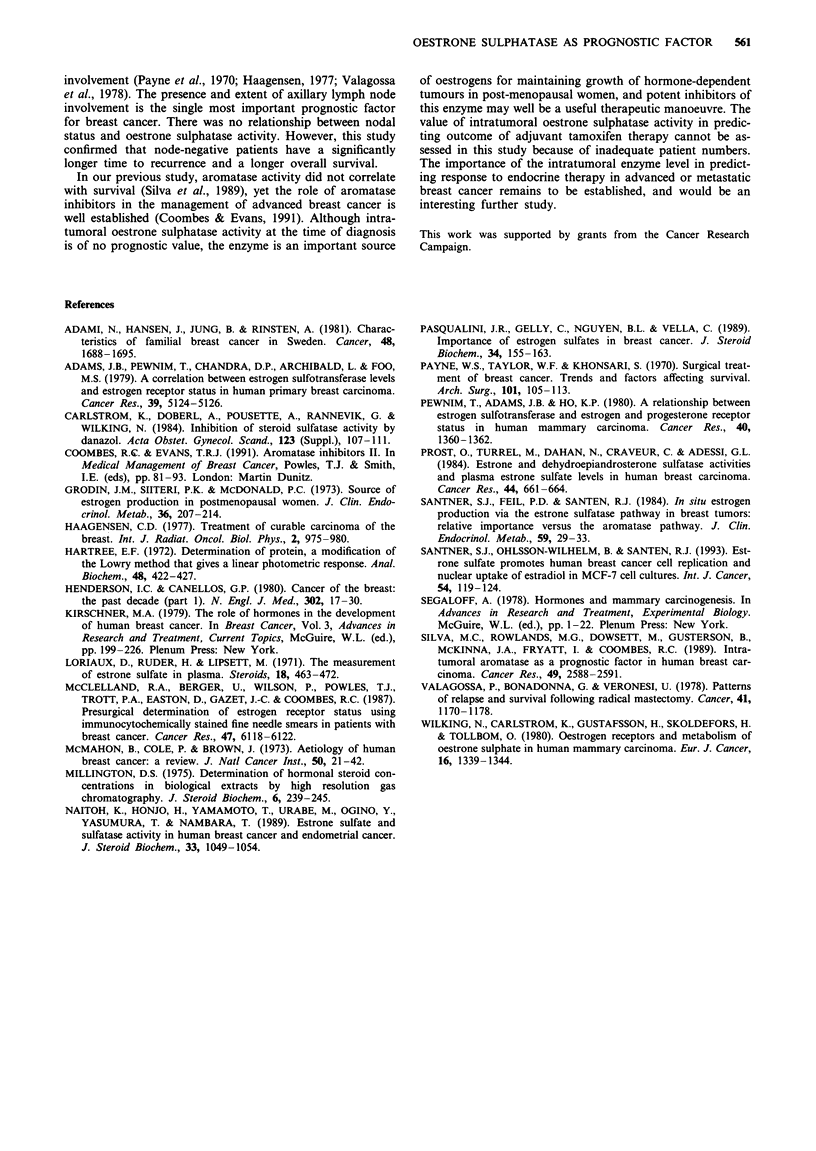# Intratumoral oestrone sulphatase activity as a prognostic marker in human breast carcinoma.

**DOI:** 10.1038/bjc.1994.101

**Published:** 1994-03

**Authors:** T. R. Evans, M. G. Rowlands, M. Law, R. C. Coombes

**Affiliations:** Department of Medical Oncology, St George's Hospital Medical School, London, UK.

## Abstract

Oestrone sulphatase is an important source of local synthesis of biologically active oestrogens in human breast cancer. The oestrone sulphatase enzyme in the particulate fraction of human breast carcinoma was characterised. The Km was 8.91 microM, and the Vmax was 0.022 nmol min-1 mg-1. Oestrone sulphatase activity was detected in 93 of 104 human breast carcinoma samples (89%), and mean activity was 0.041 nmol min-1 mg-1 (range 0-0.399 nmol min-1 mg-1). There was no significant correlation between intratumoral oestrone sulphatase activity and oestrogen receptor status, or with any other prognostic factors. Intratumoral enzyme levels were not associated with time to recurrence or with overall survival time. It thus appears that, although a useful source of intratumoral oestrogens, oestrone sulphatase activity is not of prognostic significance in breast carcinoma.


Br. J. Cancer (1994), 69, 555-561 © Macmillan Press Ltd., 1994

### Intratumoral oestrone sulphatase activity as a prognostic marker in human breast carcinoma

T.R.J. Evans[1], M.G. Rowlands[2], M. Law[2] & R.C. Coombes[3]

[1]*Department of Medical Oncology, St. George's Hospital Medical School, Cranmer Terrace, London SW17 0RE, UK;* [2]*Institute of Cancer Research, 15 Cotswold Road, Belmont, Sutton, Surrey SM2 5NG, UK;* [3]*Department of Clinical Oncology, Charing Cross Hospital, Fulham Palace Road, London W6 8RF, UK.*

**Summary** Oestrone sulphatase is an important source of local synthesis of biologically active oestrogens in human breast cancer. The oestrone sulphatase enzyme in the particulate fraction of human breast carcinoma was characterised. The $K_m$ was 8.91 μM, and the $V_{max}$ was 0.022 nmol $min^{-1}$ $mg^{-1}$. Oestrone sulphatase activity was detected in 93 of 104 human breast carcinoma samples (89%), and mean activity was 0.041 nmol $min^{-1}$ $mg^{-1}$ (range 0-0.399 nmol $min^{-1}$ $mg^{-1}$). There was no significant correlation between intratumoral oestrone sulphatase activity and oestrogen receptor status, or with any other prognostic factors. Intratumoral enzyme levels were not associated with time to recurrence or with overall survival time. It thus appears that, although a useful source of intratumoral oestrogens, oestrone sulphatase activity is not of prognostic significance in breast carcinoma.

Approximately one-third of human breast carcinomas are hormone dependent (Henderson & Canellos, 1980). Animal studies and clinical trials of anti-oestrogens and inhibitors of oestrogen biosynthesis have confirmed that oestrogens are the most important hormones involved in supporting growth of hormone-dependent breast cancers (Segaloff, 1978; Kirschner, 1979). Indeed, oestrogen deprivation by these means results in objective tumour regression.

Plasma levels of oestrone and oestradiol in post-menopausal women are very low, however the oestrogen concentration in breast carcinoma tissues is an order of magnitude higher than in the plasma (Millington, 1975), suggesting local intratumoral production of oestrogens in breast cancer cells from precursor substrates. It was originally considered that the predominant source of oestrogen production in post-menopausal women is the extra-glandular conversion of androstenedione to oestrone by peripheral tissues catalysed by the aromatase enzyme (Grodin *et al.*, 1973). However oestrone sulphate is the most abundant oestrogen in peripheral blood (Loriaux *et al.*, 1971), and plasma oestrone sulphate levels are higher in post-menopausal women with breast cancer than in normal post-menopausal women (Prost *et al.*, 1984). Studies involving MCF-7 cell lines have shown that oestrone sulphate itself is unable to bind to the oestrogen receptor (OR) and stimulate a biological response, but can be taken up by these cells and hydrolysed to unconjugated oestrone in amounts sufficient to stimulate a biological response (Pasqualini *et al.*, 1989).

Hydrolysis of oestrone sulphate to oestrone by human breast carcinoma tissue has been demonstrated by several groups (Wilking *et al.*, 1980; Carlstrom *et al.*, 1984; Prost *et al.*, 1984; Santner *et al.*, 1984; Naitoh *et al.*, 1989). Wilking *et al.* (1980) found oestrone sulphatase activity in all of 23 human breast carcinoma samples assayed; Prost *et al.* (1984) found oestrone sulphatase activity in all of 21 tumours assayed, and Santner *et al.* (1984) found oestrone sulphatase activity in all of 35 tumours assayed. There was no correlation between oestrone sulphatase activity and OR status in all three studies. In contrast, higher levels of oestrone sulphotransferase, which catalyses the formation of oestrone sulphate from oestrone, have been found in breast cancer samples which are positive for both oestrogen and progesterone receptors (Adams *et al.*, 1979; Pewnim *et al.*, 1980). Intratumoral oestrone sulphatase activity is higher than in surrounding normal breast tissue (Naitoh *et al.*, 1989).

Correspondence: T.R.J. Evans.
Received 6 May 1993; and in revised form 24 August 1993.

Moreover, a comparative study of oestrone sulphatase and aromatase activities in human breast cancer samples suggested that the oestrone sulphatase pathway is the predominant source of intratumoral oestrogen production in post-menopausal women (Santner *et al.*, 1984). Moreover, the biological effects demonstrated in response to physiological concentrations of oestrone sulphate in MCF-7 cell cultures provide further evidence of the relevance of this pathway in the production of intratumoral oestrogens in breast carcinoma tissues (Santner *et al.*, 1993).

Previous work from our laboratory has demonstrated a significant correlation between intratumoral aromatase activity and histological grade in human breast carcinoma, but no correlation either with other prognostic factors or with overall survival (Silva *et al.*, 1989). Consequently, we considered it pertinent to investigate further intratumoral oestrone sulphatase activity as a prognostic factor in human breast cancer, and its relationship to other factors believed to be of prognostic value.

### Materials and methods

### *Reagents*

[6,7-$^3$H]oestrone sulphate (specific activity 47.7 Ci $mmol^{-1}$) was purchased from New England Nuclear Division (Du Pont, UK). Purity was checked by thin-layer chromatography (TLC) (Silica gel Merck 5415 Kieselgel F254) using the following solvent system: ethyl acetate-methanol-ammonium hydroxide (75:25:2, v/v). [14-$^{14}$C]oestrone (specific activity 60 Ci $mmol^{-1}$) was purchased from Amersham International (Amersham, UK). Unlabelled oestrone sulphate was purchased from Sigma (Poole, UK).

### *Tissues and tissue preparation*

Human breast carcinoma samples were obtained from the tissue bank of the Oncology Department, St. George's Hospital Medical School, London, UK. All samples had been stored in liquid nitrogen for between 1 and 10 years.

All procedures were carried out at 0-4°C. Human breast carcinoma tissues were chopped with scissors and dissolved in 0.25 M sucrose in 50 mM Tris-HCl buffer, pH 7.4 (1 g of tissue to 10 ml of buffer), then homogenised with a Polytron for a period of 15 s. The homogenate was subjected to subcellular fractionation. The nuclear pellet was obtained by centrifugation at 1,500 *g* for 15 min, followed by centrifugation at 100,000 *g* for 70 min to separate the particulate fraction from the cytosol. All pellets were resuspended in 50 mM Tris-HCl buffer, pH 7.4, and together with the cytosols were snap frozen at −80°C and stored at −20°C. All samples were assayed for oestrone sulphatase activity and the protein content determined by the method of Hartree (1972).

*Oestrone sulphatase assay*

Before use in the assay, oestrone sulphate was purified by solvent partition with diethyl ether (5:1, v/v) in order to remove any unconjugated steroids. Radiolabelled oestrone sulphate was added to the unlabelled compound to achieve the required concentration. All assays were carried out in duplicate at 37°C in a shaking water bath. Tubes were preincubated for 1 min before initiating the reaction by addition of the tissue samples. The assay tubes (vol. 0.3 ml) contained 10 mM dithiothreitol (DTT), 1 mM EDTA, 20 μM [$^3$H]oestrone sulphate (approximately $4 \times 10^5$ c.p.m.), tissue sample and 50 mM Tris-HCl buffer at the optimal pH for the breast carcinoma tissue enzyme. Control tubes contained boiled tissue samples.

Aliquots (0.1 ml) were removed from each assay tube after 10 and 20 min incubation to ensure linearity of product formation. The reaction was terminated by addition of each aliquot to a chilled tube containing 0.1 M sodium carbonate and [14-$^{14}$C]oestrone (approximately 5,000 c.p.m.) as internal standard. The unconjugated product was separated from the substrate by adding 3 ml of ether and left to stand at room temperature. After drying with anhydrous sodium sulphate, the ether layer was separated by centrifugation and added to a scintillation vial. The sample was evaporated to dryness under nitrogen and reconstituted with 10 ml of scintillation fluid and radioactivity determined by liquid scintillation counting. The recovery of the internal standard was used to correct the amount of tritiated product formed.

The method was modified in order to identify the products of the oestrone sulphatase enzyme. The extracted ether phase was dried and centrifuged as before, an aliquot of 200 μl taken for counting and then the remainder taken to dryness by rotary evaporation and reconstituted with a small volume (25 μl) of ethyl acetate. This sample was run on a TLC plate using dichloromethane-ether (9:1, v/v) as solvent system. The plates were scanned using a Berthold LB 283 linear analyser and the radioactive peaks were scraped off the TLC plate, the silica dissolved in methanol, an aliquot retained for counting and the remainder taken to dryness under nitrogen and reconstituted in 25 μl of ethyl acetate as before. These samples were run on a TLC plate using a second solvent system, namely ethyl acetate-benzene (1:1, v/v), the plate was scanned using the Berthold LB 283 linear analyser, and the radioactive peaks identified and scraped off and dissolved in methanol before counting.

The percentage conversion of tritiated substrate to product was determined from the linear plots of product released against time, and the mean of four determinations (duplicate samples at two time points each) calculated. From this value the specific oestrone sulphatase activity for each sample was calculated. No activity was observed for the control samples.

*Oestrogen receptor content*

For many samples, the oestrogen receptor status was known from the tissue bank records. For those samples in which the OR status was not known, this was determined by an immunocytochemical method previously described (McClelland et al., 1987) using an oestrogen receptor immunocytochemistry (ERICA) kit (Abbot Laboratories).

Briefly, a frozen section of tissue was soaked in 3.7% formaldehyde/phosphate-buffered saline (PBS) for 10 min, and following rinsing in a PBS bath was placed sequentially in cold methanol (3–5 min at −20°C), cold acetone (1–3 min at −20°C) then twice in PBS at room temperature for 5 min each time. To this section was added a blocking reagent (normal goat serum) for 15 min, followed by the primary antibody for 30 min (control antibody to control sections), bridging antibody for 30 min then the PAP (peroxidase-anti-peroxidase) complex for 30 min. Two rinses of 5 min each in PBS were performed between each of these steps. Fresh chromagen was added to the sections for 6 min, then the sections were rinsed in distilled water, counterstained lightly in haematoxylin, then dehydrated and mounted. All ERICA-stained sections were analysed by one worker, and reported as either positive or negative, but not quantified.

*Statistical analyses*

One hundred and four breast carcinoma samples were assayed from patients who had undergone excisional biopsy or mastectomy between 1980 and 1989 at St. George's Hospital or at the Royal Marsden Hospital, London, UK. Medical records of these patients were reviewed retrospectively, and the following information recorded where possible: date of birth, age at menarche, parity and age at first pregnancy, age at menopause, family history of breast cancer (defined as a first-degree relative), age, weight and menopausal status at diagnosis, OR status, tumour size, time to first relapse and overall survival time.

Patients were divided into three groups of low, medium and high oestrone sulphatase activity, the cut-off points chosen to give roughly equally sized groups. These three groups were then compared in terms of the variables recorded.

All P-values quoted were calculated from tests for trend. Continuous variables (age at diagnosis, weight at diagnosis, age at menarche, age at first pregnancy and age at menopause) were analysed using linear contrasts from a simple analysis of variance. Non-parametric Kruskal-Wallis tests were performed to check the robustness of the analysis of variance to assumptions of normality. These tests gave very similar results to the parametric tests and are not presented. Categorical variables (all others) were analysed using chi-square tests for trend. Time to recurrence and overall survival were analysed using the log-rank test, survival plots being drawn using the method of Kaplan and Meier.

### Results

*Evaluation of optimal assay conditions*

Under the standard assay conditions, the control tubes containing boiled tissue gave no conversion of oestrone sulphate to oestrone. The intra-assay coefficient of variation was 7.08% ($n = 6$), the inter-assay coefficient of variation was 7.66% ($n = 8$). The characterisation of breast tumour oestrone sulphatase activity was carried out using tissue pooled from six patients to give a total of 5 g. Each result is from quadruplicate determinations ($n = 4$) and expressed as mean ± s.e. (assuming an experimental error of 15%).

Table I shows the specific activities of the sulphatase enzyme in the subcellular fractions of human breast carcinoma tissue, and the percentage that each of these fractions represents of the total activity seen in this tissue. The pooled mitochondrial/microsomal fraction (the particulate fraction) is the predominant source of enzyme activity. There was a linear relationship of increasing enzyme activity with increasing protein concentration demonstrated up to 121.5 μg of particulate fraction of breast carcinoma tissue. In addition, there was linear product formation up to 20 min, demonstrating that the enzyme was assayed under saturating conditions.

The $K_m$ and $V_{max}$ were calculated by the Lineweaver-Burk method. In the human breast carcinoma particulate fraction, the $K_m$ was 8.91 ± 1.3 μM, and $V_{max}$ 0.02 ± 0.005 nmol min$^{-1}$ mg$^{-1}$ ($n = 4$). The reciprocal plot for this enzyme was linear in nature with no evidence of substrate activation or product inhibition. The optimum pH was determined over a range of pH from 5.5 to 9.0, using 2(N-morpholino)ethane sulphonic acid (MES)–sodium hydroxide, Tris–HCl and glycine-sodium hydroxide as buffering systems. The optimum pH and buffer for the breast particulate fraction was found to be Tris–HCl, pH 7.2.

With both solvent systems only one radioactive peak was seen for the breast carcinoma enzyme product, in each case corresponding to the $R_f$ value of cold oestrone as visualised by UV light, where $R_f$ is the comparative distance of the solute and the solvent from the origin. With the dichloromethane–ether (9:1, v/v) system, the $R_f$ value was 0.39. With the ethyl acetate–benzene (1:1, v/v) system the $R_f$ value was 0.47. The $^3H/^{14}C$ ratio of the products formed remained constant after the ether extraction and after the sequential TLC analysis. This demonstrates that the only $^3H$-labelled non-polar metabolite formed is oestrone and that this is essentially pure at the ether extraction stage (Table II).

*Intratumoral oestrone sulphatase content*

Of these biopsy samples, ten patients had received local (non-endocrine) therapy between 14 and 264 months (mean 110.3 months; median 85 months) previously for a preceding primary tumour in the contralateral breast. At the time of biopsy of the second primary (from which enzyme activity was assayed), one patient had received 3 months of neoadjuvant endocrine therapy, and another patient was receiving endocrine therapy for metastatic disease. Fourteen samples were biopsies of local recurrence from primary tumours which had been treated with local (non-endocrine) therapy between 7 and 114 months (mean 23.5 months; median 37.6 months) previously, and two patients had received neoadjuvant therapy (one endocrine) prior to biopsy. Four of the patients with primary breast cancer had received neoadjuvant endocrine therapy (up to 6 months) prior to biopsy. Three biopsy specimens were from patients with metastatic disease, and who had received 3–4 endocrine treatments at the time of biopsy.

**Table I** Specific activity of oestrone sulphatase in subcellular fractions

| *Tissue fraction* | *Specific activity (nmol min$^{-1}$ mg$^{-1}$)* | *Total activity (%)* |
|---|---|---|
| *Human breast carcinoma* | | |
| Nuclear | 0.19 ± 0.04 | 18.1 |
| Mitochrondrial/microsomal | 0.89 ± 0.13 | 81.9 |
| Cytosolic | No activity | |
| Homogenate | 0.13 ± 0.02 | |

**Table II** Purity of [$^3$H]oestrone product of human breast carcinoma oestrone sulphatase

| *Sample* | *$^3H/^{14}C$ ratio* |
|---|---|
| Ether extraction | 5.64:1 |
| Dichloromethane–ether (9:1) | 5.52:1 |
| Ethyl acetate–benzene (1:1) | 5.53:1 |

**Figure 1** The distribution of intratumoral oestrone sulphatase in 104 human breast carcinoma samples.

Each result is the mean of quadruplicate determinations. As the tumour samples were small (<1 g), and as repeated freezing and thawing would lead to degradation of enzyme activity, it was not possible to determine if prolonged storage in liquid nitrogen for different periods of time would result in different enzyme activity from fresh tissue.

Intratumoral oestrone sulphatase activity was detected in 93 of 104 breast carcinoma samples assayed (89%; range 0–0.399 nmol min$^{-1}$ mg$^{-1}$). The distribution of oestrone sulphatase activity in these samples is demonstrated in Figure 1. Two patients were omitted from subsequent analyses because of insufficient clinical data. Patients were divided into roughly equal groups, low ($\leqslant$0.019 nmol min$^{-1}$ mg$^{-1}$; $n \pm 32$), medium (0.02–0.39 nmol min$^{-1}$ mg$^{-1}$; $n = 36$) or high oestrone sulphatase activity (>0.04 nmol min$^{-1}$ mg$^{-1}$; $n = 34$).

*Relationship of intratumoral oestrone sulphatase to other prognostic factors (Table III)*

*Oestrogen receptor status* Oestrogen receptor status was known in 96 of 102 cases; 44 patients were OR positive and 52 were OR negative. In the low oestrone sulphatase activity group, 15 were OR positive and 13 OR negative. In the medium activity group 16 were OR positive and 19 were OR negative. In the high-activity group 13 were OR positive and 20 were OR negative. There was no significant correlation between enzyme activity and receptor status ($P = 0.33$).

*Node status* In 87 of 102 cases the presence or absence of histologically involved regional lymph nodes was known. In 38 patients at least one lymph node was involved with tumour, but there was no lymph node involvement in 49 cases. In the low-activity group, 14 were node positive, 13 were node negative; in the medium-activity group 13 were node positive and 21 were node negative; in the high-activity group 11 were node positive and 15 were node negative. There was no significant correlation between node status and enzyme activity ($P = 0.57$).

*Age at the time of diagnosis* The age at the time of diagnosis was known in 97 of 102 cases. The mean age at diagnosis was 56 ± 12 (s.d.); the median age at diagnosis was 55 (range 29–86). In the low-activity group, the mean age was 56 ± 13, and the median age was 55 (range 36–86) for 30 patients. In the medium-activity group the mean age was 57 ± 13 years and the median age was 58.5 years (range 34–80) for 36 patients. In the high-activity group, the mean age was 54 ± 11 years and the median age was 52 years (range 29–74) for 31 patients. There was no significant correlation between oestrone sulphatase activity and the age of the patient at the time of diagnosis ($P = 0.44$).

*Histology* Histology reports were obtained from patient records of 94 patients. There were seven cases of infiltrative lobular carcinoma, five of ductal carcinoma *in situ* (DCIS) and 79 of infiltrative ductal carcinoma (IDC). There was one case each of anaplastic carcinoma, papillary carcinoma and mucinous carcinoma, and for the purposes of this study these were analysed with the IDC group. In the low-activity group, there were 27 cases of IDC, two lobular carcinomas and one DCIS. In the medium-activity group, there were 31 cases of IDC, three cases of lobular carcinoma and two of DCIS. In the high-activity group, there were 24 cases of IDC, two of lobular carcinoma and two of DCIS. The histology was unknown in two cases in the low-activity group, and in six

Table III Oestrone sulphatase activity in breast cancer

| Variable | Low (n = 32) | Medium (n = 36) | High (n = 34) | P-value | Overall (n = 102) |
|---|---|---|---|---|---|
| *Node status* | | | | | |
| + ve | 14 | 13 | 11 | 0.57 | 38 |
| − ve | 13 | 21 | 15 | | 49 |
| Not known | 5 | 2 | 8 | | 15 |
| *OR status* | | | | | |
| + ve | 15 | 16 | 13 | 0.33 | 44 |
| − ve | 13 | 19 | 20 | | 52 |
| Not known | 4 | 1 | 1 | | 6 |
| *Histology* | | | | | |
| IDC | 27 | 31 | 24 | 0.77 | 82 |
| Lobular | 2 | 3 | 2 | | 7 |
| DCIS | 1 | 2 | 2 | | 5 |
| Not known | 2 | 0 | 6 | | 8 |
| *Age at diagnosis* | | | | | |
| Mean (s.d.) | 56 (13) | 57 (13) | 54 (11) | 0.44 | 56 (12) |
| Median (range) | 55 (36–86) | 58.5 (34–80) | 52 (29–74) | | 55 (29–86) |
| Not known | 2 | 0 | 3 | | 5 |
| *Weight at diagnosis* (kg) | | | | | |
| Mean (s.d.) | 62 (13) | 64 (12) | 68 (13) | 0.14 | 65 (13) |
| Median (range) | 61 (41–89) | 61 (39–93) | 66 (50–104) | | 64 (39–104) |
| Not known | 16 | 15 | 13 | | 44 |
| *Age at menarche* | | | | | |
| Mean (s.d.) | 11.9 (2.1) | 13.3 (1.3) | 12.9 (1.7) | 0.081 | 12.8 (1.6) |
| Median (range) | 12 (7–15) | 14 (9–16) | 12 (11–17) | | 13 (7–17) |
| Not known | 15 | 1 | 13 | | 29 |
| *Parity* | | | | | |
| Yes | 22 | 27 | 24 | 0.99 | 73 |
| No | 6 | 9 | 6 | | 21 |
| Not known | 4 | 0 | 4 | | 8 |
| *Age at first pregnancy* | | | | | |
| Mean (s.d.) | 27 (6) | 27 (6) | 27 (5) | 0.97 | 27 (6) |
| Median (range) | 27 (17–39) | 26 (19–39) | 26 (19–38) | | 26 (17–39) |
| Not known | 8 | 6 | 9 | | 23 |
| *Menopausal status* | | | | | |
| Pre/peri | 13 | 13 | 14 | 0.90 | 40 |
| Post | 17 | 22 | 16 | | 55 |
| Not known | 2 | 1 | 4 | | 7 |
| *Age at menopause* | | | | | |
| Mean (s.d.) | 49 (7) | 48 (7) | 48 (6) | 0.64 | 48 (6) |
| Median (range) | 50 (35–56) | 48.5 (28–58) | 49 (35–54) | | 49 (28–58) |
| Not known | 2 | 2 | 1 | | 5 |
| *Family history* | | | | | |
| Yes | 5 | 11 | 6 | 0.94 | 22 |
| No | 21 | 23 | 21 | | 65 |
| Not known | 6 | 2 | 7 | | 15 |
| *Tumour size* | | | | | |
| T1/T2 | 10 | 17 | 9 | 0.64 | 36 |
| T3/T4 | 7 | 6 | 4 | | 17 |
| Not known | 15 | 13 | 21 | | 49 |

*P*-values calculated from tests for trend.

Table IV Prognostic significance of oestrone sulphatase level and node status

| Variable | Number | Time to recurrence Obs. | Exp. | P-value | Overall survival Obs. | Exp. | P-value |
|---|---|---|---|---|---|---|---|
| *Oestrone sulphatase* | | | | | | | |
| Low | 28 | 12 | 13.1 | | 8 | 10.2 | |
| Medium | 35 | 17 | 13.2 | 0.815 | 10 | 9.3 | 0.449 |
| High | 31 | 9 | 11.7 | | 9 | 7.6 | |
| *Node status* | | | | | | | |
| Negative | 48 | 10 | 20.0 | 0.001 | 6 | 13.1 | 0.004 |
| Positive | 36 | 22 | 12.0 | | 16 | 8.9 | |

All *P*-values calculated from log-rank test for trend.

cases in the high-activity group. There was no significant difference between these groups ($P = 0.77$).

The association of intratumoral oestrone sulphatase with the other putative prognostic factors is shown in Table III. There was no significant correlation between the enzyme level and body weight at the time of diagnosis, parity, age at first pregnancy, tumour size, menopausal status at diagnosis, age at menopause and family history of breast cancer.

*Time to disease recurrence (Table IV)*

The time to disease recurrence from primary treatment, or the time to disease progression in patients with advanced disease, was recorded. The percentage probability recurrence rate for each of 10 years of follow-up was calculated. For the low-activity group, 12 of 28 patients suffered relapse/recurrence in the follow-up period, as did 17 of 35 patients in the medium-activity group and 9 of 31 patients in the high-activity group in the same time period. The log-rank test for trend showed no significant correlation between enzyme activity and time to disease recurrence or disease relapse ($P = 0.82$). The plot of percentage probability recurrence free against years since primary treatment is shown in Figure 2.

*Overall survival time (Table IV)*

The overall survival from the time that the initial biopsy was taken (from which the oestrone sulphatase activity was determined) was recorded, that is the overall survival time from initial diagnosis or, in some cases, the overall survival time from local recurrence. In the low-activity group, 8 of 28 patients had died, in the medium-activity group 10 of 35 patients had died and in the high-activity group 9 of 31 patients had died. The percentage probability of survival for the three groups was calculated and plotted against years from primary treatment (Figure 3). The log-rank test for trend showed no significant correlation between the overall survival time and the enzyme activity level ($P = 0.45$).

*Time to recurrence by node status (Table IV)*

In this series of patients, 22 of 36 node-positive patients had suffered recurrence or relapse in the follow-up period, but only 10 of 48 node-negative patients. The percentage probability of being recurrence free was calculated and plotted against years of follow-up (Figure 4). The log-rank test for trend confirmed that node-negative patients had a significantly longer time to recurrence than node-positive patients ($P = 0.001$).

*Overall survival time by node status (Table IV)*

In the period of follow-up, 16 of 36 node-positive patients died, and only 6 of 48 node-negative patients. The percentage probability of survival was calculated and plotted against years of follow-up (Figure 5). The log-rank test for trend confirmed that node-negative patients have a significantly prolonged overall survival time compared with node-positive patients ($P = 0.004$).

*Relationship of intratumoral oestrone sulphatase activity to prognostic factors in post-menopausal women*

The above analyses were repeated for the 55 patients in this study who were post-menopausal. There was no significant correlation in this group of patients between the intratumoral oestrone sulphatase level and OR status ($P = 0.586$), node status ($P = 0.976$), age at diagnosis ($P = 0.21$), histology ($P = 0.928$) or any of the other prognostic factors studied. These results are shown in Table V.

*Relationship of intratumoral oestrone sulphatase to outcome after adjuvant tamoxifen therapy*

Only 21 patients received adjuvant tamoxifen (20 mg daily) therapy at diagnosis. The median oestrone sulphatase activity (0.041 nmol $min^{-1}$ $mg^{-1}$; range 0–0.399) was significantly higher in patients who did not relapse ($n = 12$; follow-up of 12–38 months) than in patients who relapsed ($n = 9$, follow-up of 1–28 months) after adjuvant tamoxifen therapy (mean activity 0.021; range 0–0.093 nmol $min^{-1}$ $mg^{-1}$), $P < 0.05$ (Mann–Whitney test). However this difference was no longer significant if only OR-positive patients were analysed ($n = 16$).

**Figure 2** The time to recurrence demonstrated as percentage probability recurrence free *vs* years since primary treatment for low (· · ·, 12/28), medium (——, 17/35) and high (——, 9/31) oestrone sulphatase activity.

**Figure 3** Overall survival expressed as percentage probability of survival *vs* years since primary treatment for low (· · ·, 8/28), medium (——, 10/35) and high (——, 9/31) oestrone sulphatase activity.

**Figure 4** The time to recurrence by node status (——, node negative, 10/48; ---, node positive, 22/36) expressed as percentage probability of recurrence free *vs* years since primary treatment.

**Figure 5** The overall survival by node status (——, node negative, 6/48; ---, node positive, 16/36) expressed as percentage probability of survival *vs* years since primary treatment.

**Table V** Oestrone sulphatase activity in breast cancer

| *Variable* | *Low* (n = *17)* | *Medium* (n = *22)* | *High* (n = *16)* | P-*value* | *Overall* (n = 55) |
|---|---|---|---|---|---|
| *Node status* | | | | | |
| + ve | 7 | 8 | 6 | 0.976 | 21 |
| – ve | 8 | 13 | 8 | | 28 |
| Not known | 2 | 1 | 2 | | 5 |
| *OR status* | | | | | |
| + ve | 9 | 12 | 7 | 0.586 | 28 |
| – ve | 6 | 10 | 8 | | 24 |
| Not known | 2 | 0 | 1 | | 3 |
| *Histology* | | | | | |
| IDC | 16 | 18 | 13 | 0.928 | 47 |
| Lobular | 0 | 2 | 1 | | 3 |
| DCIS | 1 | 2 | 0 | | 3 |
| Not known | 0 | 0 | 2 | | 2 |
| *Age at diagnosis* | | | | | |
| Mean (s.d.) | 66 (9) | 66 (8) | 62 (7) | 0.21 | 65 (8) |
| Median (range) | 63 (54–86) | 64.5 (50–80) | 60 (48–74) | | 63 (48–86) |
| Not known | 0 | 0 | 0 | | 0 |
| *Weight* (kg) | | | | | |
| Mean (s.d.) | 63 (11) | 67 (14) | 67 (11) | 0.415 | 66 (12) |
| Median (range) | 67 (41–75) | 67 (39–93) | 67 (53–93) | | 67 (39–93) |
| Not known | 8 | 9 | 1 | | 18 |
| *Age at menarche* | | | | | |
| Mean (s.d.) | 11.8 (5.6) | 13.5 (1.5) | 12.8 (1.8) | 0.139 | 12.8 (1.9) |
| Median (range) | 12.5 (7–14) | 14 (9–16) | 12 (11–17) | | 13.5 (7–17) |
| Not known | 5 | 1 | 5 | | 11 |
| *Tumour size* | | | | | |
| T1/T2 | 6 | 9 | 5 | 0.767 | 20 |
| T3/T4 | 4 | 3 | 2 | | 9 |
| Not known | 7 | 10 | 9 | | 26 |
| *Parity* | | | | | |
| Yes | 12 | 16 | 12 | 0.916 | 40 |
| No | 4 | 6 | 3 | | 13 |
| Not known | 1 | 0 | 1 | | 2 |
| *Age at first pregnancy* | | | | | |
| Mean (s.d.) | 29 (6) | 28 (7) | 28 (7) | 0.602 | 28 (6) |
| Median (range) | 30 (21–39) | 26 (19–39) | 25.5 (19–38) | | 26.5 (19–39) |
| Not known | 9 | 10 | 6 | | 25 |
| *Age at menopausal* | | | | | |
| Mean (s.d.) | 49 (8) | 48 (7) | 48 (6) | 0.518 | 48 (7) |
| Median (range) | 51.5 (35–56) | 48.5 (28–58) | 49.5 (35–54) | | 49 (28–58) |
| Not known | 5 | 2 | 2 | | 9 |
| *Family history* | | | | | |
| Yes | 2 | 5 | 4 | 0.447 | 11 |
| No | 13 | 15 | 10 | | 38 |
| Not known | 2 | 2 | 2 | | 6 |

All *P*-values calculated from tests from trend.

### Discussion

The cause of breast cancer is not known. However epidemiological evidence suggests that increased incidence is associated with endocrine, environmental and genetic factors. Early menarche, age at the menopause, age at first pregnancy and the parity of the patient all show correlation with incidence of breast cancer (McMahon *et al.*, 1973). In addition, women with a family history of breast cancer are at increased risk of developing breast cancer, with a relative risk of 1.7–2.5 for women with a first-degree relative with breast cancer (Adami *et al.*, 1981). However, there is no significant correlation between intratumoral oestrone sulphatase activity and age at onset of menarche, age at onset of menopause, parity of the patients, age at first pregnancy, family history and menopausal status of the patient at the time of diagnosis. Moreover, this study confirms the earlier reports which suggested that there was no significant correlation between enzyme activity and the presence of the oestrogen receptor. Although there is an association between aromatase activity and histological grade (Silva *et al.*, 1989), there is no relationship between oestrone sulphatase activity and histological subtype, although information on the grade of histology was not available in this study. However, it should be noted that most tumours in this study were IDC. For those tumours at the time of diagnosis, and for those advanced or recurrent tumours at the time of biopsy, there was no association between enzyme activity and either time to recurrence (or to disease progression) or overall survival time. Nor was there significant correlation of enzyme activity with any of the prognostic factors studied when the data were analysed for post-menopausal women alone. However, only 55 patients were post-menopausal, and this number is too small to allow survival curves to be calculated for this subgroup alone. Furthermore, as this study was performed with only 102 samples, there is insufficient power to detect modest associations as statistically significant. However, such modest associations are unlikely to be of significance in clinical practice.

Patients with histologically negative axillary lymph node involvement have a significantly greater probability of survival than patients with positive histological lymph node involvement (Payne *et al.*, 1970; Haagensen, 1977; Valagossa *et al.*, 1978). The presence and extent of axillary lymph node involvement is the single most important prognostic factor for breast cancer. There was no relationship between nodal status and oestrone sulphatase activity. However, this study confirmed that node-negative patients have a significantly longer time to recurrence and a longer overall survival.

In our previous study, aromatase activity did not correlate with survival (Silva *et al.*, 1989), yet the role of aromatase inhibitors in the management of advanced breast cancer is well established (Coombes & Evans, 1991). Although intratumoral oestrone sulphatase activity at the time of diagnosis is of no prognostic value, the enzyme is an important source of oestrogens for maintaining growth of hormone-dependent tumours in post-menopausal women, and potent inhibitors of this enzyme may well be a useful therapeutic manoeuvre. The value of intratumoral oestrone sulphatase activity in predicting outcome of adjuvant tamoxifen therapy cannot be assessed in this study because of inadequate patient numbers. The importance of the intratumoral enzyme level in predicting response to endocrine therapy in advanced or metastatic breast cancer remains to be established, and would be an interesting further study.

This work was supported by grants from the Cancer Research Campaign.

### References

ADAMI, N., HANSEN, J., JUNG, B. & RINSTEN, A. (1981). Characteristics of familial breast cancer in Sweden. *Cancer*, **48**, 1688–1695.

ADAMS, J.B., PEWNIM, T., CHANDRA, D.P., ARCHIBALD, L. & FOO, M.S. (1979). A correlation between estrogen sulfotransferase levels and estrogen receptor status in human primary breast carcinoma. *Cancer Res.*, **39**, 5124–5126.

CARLSTROM, K., DOBERL, A., POUSETTE, A., RANNEVIK, G. & WILKING, N. (1984). Inhibition of steroid sulfatase activity by danazol. *Acta Obstet. Gynecol. Scand.*, **123** (Suppl.), 107–111.

COOMBES, R.C. & EVANS, T.R.J. (1991). Aromatase inhibitors II. In *Medical Management of Breast Cancer*, Powles, T.J. & Smith, I.E. (eds), pp. 81–93. London: Martin Dunitz.

GRODIN, J.M., SIITERI, P.K. & MCDONALD, P.C. (1973). Source of estrogen production in postmenopausal women. *J. Clin. Endocrinol. Metab.*, **36**, 207–214.

HAAGENSEN, C.D. (1977). Treatment of curable carcinoma of the breast. *Int. J. Radiat. Oncol. Biol. Phys.*, **2**, 975–980.

HARTREE, E.F. (1972). Determination of protein, a modification of the Lowry method that gives a linear photometric response. *Anal. Biochem.*, **48**, 422–427.

HENDERSON, I.C. & CANELLOS, G.P. (1980). Cancer of the breast: the past decade (part 1). *N. Engl. J. Med.*, **302**, 17–30.

KIRSCHNER, M.A. (1979). The role of hormones in the development of human breast cancer. In *Breast Cancer*, Vol. 3, *Advances in Research and Treatment, Current Topics*, McGuire, W.L. (ed.), pp. 199–226. Plenum Press: New York.

LORIAUX, D., RUDER, H. & LIPSETT, M. (1971). The measurement of estrone sulfate in plasma. *Steroids*, **18**, 463–472.

MCCLELLAND, R.A., BERGER, U., WILSON, P., POWLES, T.J., TROTT, P.A., EASTON, D., GAZET, J.-C. & COOMBES, R.C. (1987). Presurgical determination of estrogen receptor status using immunocytochemically stained fine needle smears in patients with breast cancer. *Cancer Res.*, **47**, 6118–6122.

MCMAHON, B., COLE, P. & BROWN, J. (1973). Aetiology of human breast cancer: a review. *J. Natl Cancer Inst.*, **50**, 21–42.

MILLINGTON, D.S. (1975). Determination of hormonal steroid concentrations in biological extracts by high resolution gas chromatography. *J. Steroid Biochem.*, **6**, 239–245.

NAITOH, K., HONJO, H., YAMAMOTO, T., URABE, M., OGINO, Y., YASUMURA, T. & NAMBARA, T. (1989). Estrone sulfate and sulfatase activity in human breast cancer and endometrial cancer. *J. Steroid Biochem.*, **33**, 1049–1054.

PASQUALINI, J.R., GELLY, C., NGUYEN, B.L. & VELLA, C. (1989). Importance of estrogen sulfates in breast cancer. *J. Steroid Biochem.*, **34**, 155–163.

PAYNE, W.S., TAYLOR, W.F. & KHONSARI, S. (1970). Surgical treatment of breast cancer. Trends and factors affecting survival. *Arch. Surg.*, **101**, 105–113.

PEWNIM, T., ADAMS, J.B. & HO, K.P. (1980). A relationship between estrogen sulfotransferase and estrogen and progesterone receptor status in human mammary carcinoma. *Cancer Res.*, **40**, 1360–1362.

PROST, O., TURREL, M., DAHAN, N., CRAVEUR, C. & ADESSI, G.L. (1984). Estrone and dehydroepiandrosterone sulfatase activities and plasma estrone sulfate levels in human breast carcinoma. *Cancer Res.*, **44**, 661–664.

SANTNER, S.J., FEIL, P.D. & SANTEN, R.J. (1984). *In situ* estrogen production via the estrone sulfatase pathway in breast tumors: relative importance versus the aromatase pathway. *J. Clin. Endocrinol. Metab.*, **59**, 29–33.

SANTNER, S.J., OHLSSON-WILHELM, B. & SANTEN, R.J. (1993). Estrone sulfate promotes human breast cancer cell replication and nuclear uptake of estradiol in MCF-7 cell cultures. *Int. J. Cancer*, **54**, 119–124.

SEGALOFF, A. (1978). Hormones and mammary carcinogenesis. In *Advances in Research and Treatment, Experimental Biology*. McGuire, W.L. (ed.), pp. 1–22. Plenum Press: New York.

SILVA, M.C., ROWLANDS, M.G., DOWSETT, M., GUSTERSON, B., MCKINNA, J.A., FRYATT, I. & COOMBES, R.C. (1989). Intratumoral aromatase as a prognostic factor in human breast carcinoma. *Cancer Res.*, **49**, 2588–2591.

VALAGOSSA, P., BONADONNA, G. & VERONESI, U. (1978). Patterns of relapse and survival following radical mastectomy. *Cancer*, **41**, 1170–1178.

WILKING, N., CARLSTROM, K., GUSTAFSSON, H., SKOLDEFORS, H. & TOLLBOM, O. (1980). Oestrogen receptors and metabolism of oestrone sulphate in human mammary carcinoma. *Eur. J. Cancer*, **16**, 1339–1344.